# Recommendations for clinical monitoring of patients with acid sphingomyelinase deficiency (ASMD)

**DOI:** 10.1016/j.ymgme.2018.11.014

**Published:** 2018-11-29

**Authors:** Melissa Wasserstein, Carlo Dionisi-Vici, Roberto Giugliani, Wuh-Liang Hwu, Olivier Lidove, Zoltan Lukacs, Eugen Mengel, Pramod K. Mistry, Edward H. Schuchman, Margaret McGovern

**Affiliations:** a Children’s Hospital at Montefiore, Albert Einstein College of Medicine, Bronx, NY, USA; b Division of Metabolism, Bambino Gesù Childrens Hospital, Rome, Italy; c Medical Genetics Service, HCPA, Department of Genetics, UFRGS, Porto Alegre, Brazil; d National Taiwan University Hospital, Taipei, Taiwan; e Groupe Hospitalier Diaconesses-Croix St Simon, Paris, France; f University Medical Center Hamburg-Eppendorf, Hamburg, Germany; g University Medical Center, Johannes Gutenberg University, Mainz, Germany; h Yale University, New Haven, CT, United States; i Icahn School of Medicine at Mount Sinai, New York, NY, USA; j Stony Brook University, School of Medicine, Stony Brook, NY, USA

**Keywords:** Acid sphingomyelinase deficiency, ASMD, Patient monitoring

## Abstract

**Background:**

Acid sphingomyelinase deficiency (ASMD), a rare lysosomal storage disease, results from mutations in *SMPD1*, the gene encoding acid sphingomyelinase (ASM). As a result, sphingomyelin accumulates in multiple organs including spleen, liver, lung, bone marrow, lymph nodes, and in the most severe form, in the CNS and peripheral nerves. Clinical manifestations range from rapidly progressive and fatal infantile neurovisceral disease, to less rapidly progressing chronic neurovisceral and visceral forms that are associated with significant morbidity and shorter life span due to respiratory or liver disease.

**Objectives:**

To provide a contemporary guide of clinical assessments for disease monitoring and symptom management across the spectrum of ASMD phenotypes.

**Methods:**

An international group of ASMD experts in various research and clinical fields used an evidence-informed consensus process to identify optimal assessments, interventions, and lifestyle modifications.

**Results:**

Clinical assessment strategies for major organ system involvement, including liver, spleen, cardiovascular, pulmonary, and neurological/developmental are described, as well as symptomatic treatments, interventions, and/or life style modifications that may lessen disease impact.

**Conclusions:**

There is currently no disease-specific treatment for ASMD, although enzyme replacement therapy with a recombinant human ASM (olipudase alfa) is in clinical development. Current monitoring addresses symptoms and multisystem involvement. Recommended interventions and lifestyle modifications are designed to address morbidity and disease complications and improve patient quality of life. While infantile neurovisceral ASMD is uniformly fatal in early childhood, patients with chronic visceral and chronic neurovisceral ASMD require appropriate management throughout childhood and adulthood by an interdisciplinary clinical team.

## 1. Introduction

Acid sphingomyelinase deficiency (ASMD), also known as Niemann Pick disease type A and type B, is a rare lysosomal storage disorder resulting from deficiency of the lysosomal enzyme acid sphingomyelinase (ASM) due to bi-allelic mutations in the sphingomyelin phosphodiesterase 1 gene, *SMPD1* [1,2]. ASMD results primarily in the progressive accumulation of sphingomyelin within the mononuclear phagocytic system and hepatocytes, and manifests as a multi-system disease involving the spleen, liver, lung, bone marrow, and lymph nodes, and in severe forms of the disease, the CNS and peripheral nervous system. While the same metabolic defect is common to all ASMD patients, disease severity is determined by the presence or absence of neurological involvement, the extent of systemic disease, and the rate of disease progression, resulting in a wide spectrum of clinical manifestations. Infantile neurovisceral ASMD [Niemann-Pick disease type A (NPD A)] is the most severe form and is rapidly progressive and uniformly fatal in early childhood [3]. More slowly progressive chronic neurovisceral ASMD (intermediate, NPD A/B, NPD B variant) and chronic visceral ASMD (NPD B) have onset of symptoms in childhood through adulthood and are associated with significant morbidity [4], and reduced life expectancy due to respiratory and/or liver disease [5,6]. While the epidemiology of ASMD varies by region, chronic forms of ASMD comprise the greatest number of cases [7]. The features of the subtypes reflecting the clinical spectrum of ASMD are described in Table 1.

The spectrum of clinical manifestations that overlap with other disorders can make diagnosis a challenge, and in the case of chronic forms can lead to a long diagnostic odyssey for patients. Diagnostic guidelines have been published for infantile neurovisceral ASMD, chronic neurovisceral and chronic visceral disease presenting in childhood, and later-onset chronic visceral ASMD presenting after childhood, based on the most common presenting ASMD symptoms, the associated symptoms predictive of ASMD, differential diagnoses, and diagnostic testing paradigms [8]. Organomegaly is the most common presenting symptom in children with all forms of ASMD, and in conjunction with the typical symptoms associated with ASMD should trigger testing for ASM enzyme activity. Gene sequencing is recommended after detecting reduced ASM activity to identify mutations that might fall within the known categories of genotype/phenotype correlations [9]. However, when rare or novel *SMPD1* mutations are identified, accurate prediction of phenotypes and/or prognosis is not feasible. In such cases, ongoing clinical assessments are necessary to determine phenotype and prognosis.

At present, there are no approved treatments for ASMD, although enzyme replacement therapy (ERT) with olipudase alfa, a recombinant human ASM, is in clinical development for the treatment of non-neurologic manifestations of ASMD [10]. Thus, current management of ASMD is targeted towards lessening the impact of individual symptoms with treatments, interventions, and lifestyle modifications designed to reduce morbidity and disease complications, and improve patient quality of life. Medical management for patients with ASMD is typically provided by metabolic disease specialists. However, primary care providers and other specialists (e.g., pediatricians, cardiologists, pulmonologists, hepatologists, and hematologists) are likely to be part of a team approach to patient care. Since the liver is a prominently affected organ in the chronic forms of ASMD, hepatologists or gastroenterologists may be the first specialists to see pediatric patients with chronic visceral and chronic neurovisceral ASMD, while some individuals with chronic visceral ASMD may not receive a diagnosis until they present with respiratory disease in late adulthood. Consultation and communication between physicians involved in the care of a patient with ASMD is important to ensure familiarity with the routine assessments necessary to manage the multisystem impact of the disease.

## 2. Objectives

This review presents global expert opinion consensus on current practices for disease monitoring in patients across the spectrum of ASMD phenotypes. The objective of this paper is to review the routine clinical assessments necessary for monitoring the multisystemic manifestations across the spectrum of ASMD phenotypes. Furthermore, suggestions for appropriate research-based follow-up are offered in anticipation of the introduction of enzyme replacement therapy. These recommendations are not intended to replace the clinical judgment of the treating physicians, or to be inflexible, but rather to provide general approaches applicable to most patients suffering from ASMD.

## 3. Methods and process

Recommendations for clinical assessments and monitoring disease status of patients with ASMD were developed by an evidence-informed consensus process. An inherent challenge in compiling these recommendations was the lack of a complete published evidence base given the rarity of the disease, therefore personal clinical experience was used to supplement published evidence. An international group of experts in the clinical and laboratory evaluation, diagnosis, treatment/management, and genetic aspects of ASMD met for 2 days in 2015 to review their collective experience with patients with ASMD and develop recommendations for monitoring of patients across the multiple ASMD phenotypes. Following the meeting, authors held teleconferences to develop the consensus document. To further assess the validity of the recommendations, the authors conferred with specialists in cardiology, hepatology, pulmonology and hematology (identified in the acknowledgment section) for critical review of the manuscript.

### 3.1. Monitoring strategies for patients with ASMD

Monitoring strategies are presented in the following sections, as well as descriptions of treatments, interventions, and/or life style modifications that may lessen the impact of symptoms. Suggested assessments are summarized in the boxes that accompany each section. This schedule of assessments addresses key ASMD-related disease manifestations to monitor over the course of the disease in childhood and adulthood. Following assessments performed upon initial diagnosis, the actual frequency of assessments should be decided by clinical teams in an individualized fashion for each patient.

### 3.2. Patients with chronic visceral and neurovisceral ASMD

The presentation, severity, and disease progression in chronic ASMD are heterogeneous [Table 1; see [4] for a review of disease manifestations and burden across ASMD phenotypes], underscoring the need for individualized management.

This section is organized by organ system with the assumption that thorough neurological and visceral clinical assessments are performed at the time of diagnosis. The schedule of assessments addresses key ASMD-related disease manifestations that require monitoring over the course of the disease. The schedule of recommended assessments for pediatric and adult patients is shown in Table 2.

### 3.3. Hepatic disease

Hepatomegaly is a near-constant feature of ASMD resulting from massive infiltration of hepatic macrophages (Kupffer cells) laden with sphingomyelin into hepatic sinusoidal spaces (classically referred to as foam cells by histology) as well as lysosomal sphingomyelin accumulation in hepatocytes. Hepatic disease is a major cause of morbidity, and one of the most common causes of death in patients with chronic visceral disease [4–6]. In an analysis of morbidities contributing to death among 85 patients with chronic ASMD, liver disease was equally prevalent in patients that died in childhood or adulthood [5]. There remains a lack of awareness that adults with chronic ASMD disease are at significant risk of cirrhosis, portal hypertension and variceal bleeding. Nearly all adult patients (*n*= 17) with ASMD screened for enrollment in a phase 1 clinical trial had some degree of hepatic fibrosis, ranging from early stage fibrosis to established cirrhosis [11]. Sphingomyelin accumulation was prominent in Kupffer cells as well as hepatocytes of patients that developed fibrosis.

At initial evaluation of liver involvement, liver panels should include tests for ferritin, iron saturation, and viral and autoimmune serology to rule out potential co-morbidities. Periodic (every 6–12 months) liver panels (i.e., transaminases, GGT, bilirubin, albumin, and prothrombin time/INR) can assist in assessment of liver function, although these tests commonly underestimate the extent of liver disease and fibrosis. Hepatocellular synthetic function assessed by INR and albumin levels may be preserved even in setting of advanced cirrhosis. While elevated hepatic transaminases are seen in most patients, liver enzyme levels may be normal or only slightly elevated in early stages of liver fibrosis. Accurate assessment of progression of liver fibrosis is important in managing ASMD patients. This can now be performed noninvasively by ultrasound-based transient elastography to measure liver stiffness. This has been validated to reliably detect stage 2 vs stage 3 fibrosis vs stage 4 fibrosis in the general population in which it has also been shown to predict complications of liver disease as well as predict the occurrence or appearance of liver lesions. Elastography readings of < 20 kPa combined with normal platelet counts of > 150,000/mm^3^ are unlikely to have high-risk varices, and patients can avoid endoscopies (reviewed in [12]). Expanded criteria have been validated, and the recommended thresholds for performing upper GI endoscopy to screen for varices are when elastography exceeds 25 kPa and platelets fall below 110,000/mm^3^ [13]. As liver stiffness values approach stage 4 fibrosis range, it should trigger upper endoscopy to screen for varices and initiate prophylactic non-selective beta blocker therapy to prevent variceal hemorrhage. In patient showing liver stiffness in stage 4 range, further assessment for degree of portal hypertension can be made noninvasively to assess for degree of splenomegaly or thrombocytopenia, however, the gold-standard for accurate assessment of portal pressures is via transjugular hepatic venous-portal vein pressure gradient measurements in selected patients. An early clue to development of portal hypertension may come from acceleration of splenomegaly. Additional monitoring of status of liver cirrhosis using scoring systems such as Child-Pugh classification and the Model for End-Stage Liver Disease (MELD), while typically used to assess patients for liver transplantation, may provide additional parameters to monitor liver disease in ASMD [12,14]. However, caution should be exercised in over-reliance on this system as it is heavily based on elevated bilirubin, INR and creatinine, indicators that generally do not accurately reflect the extent of liver disease in ASMD.

Treatment options for liver disease are limited. Patients with liver disease should refrain from alcohol use. Symptom management in patients with end-stage liver disease should focus on screening for liver lesions, control of edema and ascites, prevention of bleeding due to esophageal varices with non-selective beta blockers with or without endoscopic variceal ligation according to hepatology guidelines, prevention/management of hepatic encephalopathy, vaccinations, prophylactic antibiotics for prevention of spontaneous bacterial peritonitis as appropriate, and nutritional maintenance. Liver transplantation has been performed in several ASMD patients ([15], MW personal communication), and decisions to transplant will be driven by the degree of complications resulting from cirrhosis.

### 3.4. Splenomegaly

The most common initial clinical presentation for patients with chronic ASMD is organomegaly in early childhood, although in mild disease splenomegaly may not be obvious [4]. Almost every patient described in observational studies of patients with chronic ASMD harbor splenomegaly [5,16–18]. Splenomegaly is initially caused by infiltration of lipid-laden macrophages [19], but more rapid progression of splenomegaly should raise concerns about contributing effects of portal hypertension. Splenomegaly is significant and can be massive reaching spleen volumes > 20 multiples of normal, leading to hypersplenism with increased risks for bleeding and splenic rupture, especially in the context of falling platelet counts.

There are no satisfactory treatment options for splenomegaly. Splenectomy is generally contraindicated due to the potential for exacerbation of liver disease and increased sphingomyelin accumulation in the lungs with progressive respiratory insufficiency (authors’ clinical experience). If splenectomy needs to be considered due to massive splenomegaly, pressure symptoms, and severe unsustainable hypersplenism, then partial splenectomy or partial splenic arterial embolization are potential options, which have been used in Gaucher disease [20,21]. If emergency surgery is necessary because of splenic trauma, necrosis, or rupture, partial or total splenectomy should be performed according to surgical indications with use of standard post-surgical antibiotic prophylaxes and vaccinations. Lifestyle modifications include reduction and caution in contact sports to minimize trauma to the spleen.

### 3.5. Pulmonary disease

Pulmonary involvement is a key feature of the multisystem manifestation of sphingomyelin storage [22], and most patients (> 90%) with chronic visceral ASMD have radiographic evidence of infiltrative lung disease [23,24]. The pathophysiology of pulmonary disease involves the accumulation of lipid-laden macrophages in the alveolar septa, bronchial walls, and pleura, resulting in progressive restriction of lung volumes and impaired gas exchange [25] [6]. An inflammatory component that drives the recruitment of macrophages to the lung may also be a factor as observed in the ASM knockout mouse model [19]. Interestingly, lung-only involvement, in the absence of organomegaly, has been reported in adult ASMD patients [26]. Periodic patient assessments (yearly or as dictated by symptoms) include pulmonary function testing, including assessment of the diffusing capacity of the lung (DLco), O_2_ saturation, and assessments of exercise tolerance with the modified Medical Research Council scale for assessment of dyspnea and the 6-min walk test. Although patients may have no overt respiratory symptoms, chest radiography shows typical reticulonodular patterns of infiltration. It should be noted that frequently there is a dissociation between the extent of infiltrative lung disease assessed by imaging and the degree of lung compromise indicated by pulmonary function test parameters [23]. Imaging (with chest x-ray or low-radiation HRCT) to monitor disease progression should be performed every 2–4 years.

Treatment options for pulmonary involvement in ASMD are limited. Lung lavage has been attempted, but has not been shown to be effective in alleviating symptoms and has been associated with procedure-related complications [27]. Oxygen therapy and use of bronchodilator may be needed for symptomatic pulmonary disease. Pulmonary infections are common and should be managed appropriately, including administration of preventative vaccinations for pediatric and adult patients. First-and second-hand exposure to tobacco smoke, and use of electronic cigarettes should be avoided.

### 3.6. Cardiovascular disease

Cardiac dysfunction is common in patients with advanced ASMD, and may result independent of, or as a consequence of, pulmonary disease. In a cross-sectional natural history study of 59 patients, electrocardiogram and echocardiogram abnormalities were common (28% and 50% of patients, respectively) [17]. In 103 patients with chronic ASMD, 9% had valve abnormalities [6]. Plasma lipid abnormalities are also common in ASMD. The majority of patients have an atherogenic lipid profile with low HDL cholesterol (HDLeC) and elevated LDL-C, VLDL-C, and triglyceride levels [10,18,28,29]. HDL-C levels are decreased in patients of all ages. Hypertrophy of medial and intimal smooth muscle cells has been described in distal branches of coronary arteries, and infiltration of lipid-laden macrophages may contribute to this apparent accelerated atherosclerosis [29].

### 3.7. Cardiac dysfunction

The frequency of cardiac assessments should be dictated by symptoms and findings following baseline assessments. Cardiac dysfunction occurs at an early age and monitoring is necessary in children with ASMD. Cardiac valve abnormalities are common [17] and may be mild and clinically insignificant. However, reports of severe mitral insufficiency and aortic stenosis have been reported in both children and adult patients [30,31]. Electrocardiograms should be performed annually and echocardiograms to assess valvular defects should be performed every 2–4 years. Cardiac dysfunction, including narrowing of coronary arteries due to hypertrophy of medial and intimal smooth muscle cells (independent of pulmonary disease and presumably due to sphingomyelin-laden lysosomes), has been described in adult patients with chronic ASMD [29]. Baseline assessment of coronary artery status by HRCT or cardiac computed tomography for coronary calcium scoring at age 18 is recommended, followed by scans every 2–4 years. These assessments should be considered at the same time as pulmonary assessments.

Valvular insufficiency may be treated with medications according to standard guidelines, or with surgery to repair or replace defective heart valves. Stenting and/or coronary artery bypass grafting (CABG) should also be considered for cardiovascular disease as indicated. Surgical interventions must be evaluated based on potential risks of bleeding issues or other contraindications.

### 3.8. Dyslipidemia

Annual monitoring of lipid profiles is recommended. Treatment and lifestyle changes include modifying diet for adolescents and adults, and lipid-lowering therapy for dyslipidemia according to current guidelines, and at the discretion of the treating physicians. If statins are prescribed, more frequent monitoring of liver function may be needed. Performing HRCTs for coronary calcium scoring is advocated based on an atherogenic lipoprotein profile including strikingly low HDL cholesterol [28]. In children with ASMD with significant growth restriction, dietary fat and cholesterol are often not restricted in order to ensure adequate caloric intake and optimal growth.

### 3.9. Hematology

In prospective natural history studies, thrombocytopenia and bleeding were common in patients with chronic forms of ASMD: up to 54% of 29 patients from a longitudinal study had thrombocytopenia [18], and bleeding events were reported in 49% of 59 patients from a cross-sectional study [17]. Thrombocytopenia and leucopenia tend to increase with patient age [18]. Hemorrhage can result from injury and can occur postoperatively (e.g., following routine tonsillectomy, dental surgery). Many patients have recurrent nosebleeds, sometimes requiring cauterization. More serious bleeding events including subdural hematoma, hematemesis, and hemothorax have been described [17]. Recurrent bleeding events correlate with degree of splenomegaly, but not strictly with decreased platelet counts suggesting platelet dysfunction may play a role in bleeding diathesis [17]. Unrelated but common causes of bleeding (e.g., Von Willebrand disease) should be ruled out.

Assessments for monitoring hematologic abnormalities include annual coagulation profiles and CBCs, but the frequency should be determined by the findings in individual patients and extent of involvement of other organ systems (e.g., functional status of liver). Transfusions may be needed in extreme cases of bleeding, but more commonly, interventions (e.g., nasal packing, cauterization) are needed for patients with frequent or severe nose bleeds.

### 3.10. Skeletal manifestations and growth delay

A natural history study of patients with chronic visceral ASMD showed that complaints of joint and limb pain are common in pediatric and adult patients [17]. In a study of 46 patients with chronic ASMD, children with ASMD showed lower bone mineral content (BMC) and bone mineral density (BMD) in spine, hip, and femur compared to agematched healthy subjects. The majority of adult ASMD patients have osteopenia or osteoporosis [32]. Bone fractures were reported for patients with chronic ASMD in two studies (59 patients and 46 patients, respectively) [17,32]. Twenty-five percent of children with ASMD and 53% of adults reported skeletal fractures, in some cases occurring following minimal or no trauma [32].

Growth delays are also reported in children and adolescents with chronic visceral ASMD, with delayed skeletal age, short stature, and low weight [17,33]. The majority of adolescent patients assessed in a cross-sectional study had delayed growth, bone age, and delayed puberty [17].

Weight and linear growth should be monitored in children with ASMD at least every 6–12 months, and nutritional assessments are important to ensure sufficient caloric intake for growth. The use of growth hormone therapy in ASMD has not been systematically studied, but monitoring of hormone levels may be informative in some patients.

Skeletal health in children (from infancy to adolescence) and adults should be assessed using the guidance provided by the International Society for Clinical Densitometry (ISCD) [34,35]. Assessment of BMC and BMD using dual energy X-ray absorptiometry (DXA) can be performed every 2–4 years in patients with signs of low bone density (e.g., fractures). The diagnosis of osteoporosis in children and adolescents should not be made on the basis of densitometry alone. Diagnosis of osteoporosis is indicated by history of vertebral compression (crush) fractures, or the presence of both a clinically significant long-bone fracture history and BMD *Z*-score ≤ −2.0 [34]. BMC and BMD data should be adjusted in children with growth delay. In adults, skeletal sites to assess BMD and BMC are the spine and hip, and WHO guidelines for osteoporosis diagnosis are used [35].

Standard dietary and lifestyle interventions to prevent/delay bone loss are recommended. Weight bearing exercise is recommended to prevent/delay bone loss. Physical therapy is recommended for joint and limb pain. While bisphosphonates have been used in Gaucher disease to improve bone density [36], their use in ASMD has not been studied because they inhibit ASM activity [37].

### 3.11. Neurological manifestations/cognitive deficits

While neurological symptoms are predominant in patients with infantile neurovisceral ASMD, they are variable in patients with chronic disease as summarized in Table 1. Patients with chronic visceral ASMD typically have no neurological or cognitive deficits, while patients with chronic neurovisceral ASMD display a range of symptoms ranging from mild hypotonia and hyporeflexia to more severe involvement including loss of motor function and cognitive decline. The onset of neurological symptoms in patients with chronic neurovisceral ASMD occurs later in childhood than in patients with infantile neurovisceral disease, and neurological symptoms do not progress as rapidly. Extensive neurological examinations in 64 patients with chronic ASMD identified 30% of patients (*n*= 19) with some form of neurological impairment [38]. Five of these 19 patients were diagnosed with ASMD before 5 years of age and had achieved normal developmental milestones at 2 years of age. Onset of neurological abnormalities in the 5 patients occurred between 3 and 7 years of age, with symptom severity that ranged from moderate to severe. Loss of milestones and neurodegeneration were progressive in the 5 patients, and all had abnormal retinal findings and peripheral neuropathy. The Q292K mutation (also referred to as Q294K), which has been linked to neurological involvement in patients with chronic ASMD [39], was present in 1 or 2 copies in each patient. In the remaining 14/19 patients, neurological abnormalities were minor and non-progressive, and consisted primarily of hypotonia, ataxia, or hyporeflexia [38].

Assessments for neurological and cognitive deficits include baseline clinical neurological and ophthalmology examinations, including direct ophthalmoscopy to assess presence of cherry red spots. Age-appropriate neuropsychology assessments should be performed periodically in pediatric and adolescent patients if deemed necessary. Patients of all ages should be monitored for development of peripheral neuropathy. Patients with 1 or 2 copies of the Q292K mutation should be monitored more frequently during childhood due to the association of this mutation with more severe neurologic abnormalities. Interventions include educational support and physical therapy, as needed.

### 3.12. Management of pregnancy

There are limited reports on the impact of chronic visceral ASMD on pregnancy. Women with chronic visceral ASMD and exhibiting a wide range of somatic manifestations, including significant pulmonary disease and hepatosplenomegaly, appear to have normal pregnancies and healthy newborns (McGovern, personal observations). Esophageal varices contribute to increased bleeding risks as pregnancy progresses and during the perinatal period. Prenatal care by a high-risk obstetrician is indicated to monitor pulmonary and hematologic status.

### 3.13. Patients with infantile neurovisceral ASMD

Infantile neurovisceral ASMD is a uniformly fatal disease, with the majority of patients dying by 3 years of age [3,16]. Clinical assessments, including liver function, lung function, and hematology, are usually made around the time of diagnosis, but invasive assessments are generally not necessary unless they can assist in improving patient quality of life. For example, noninvasive head circumference measurements can suggest hydrocephalus if a rapid increase in size is detected. Abdominal imaging is not usually necessary, as progressive hepatosplenomegaly is predicted and imaging studies do not contribute to the clinical management of patients with severe ASMD. Blood testing should be considered at the discretion of the physician (e.g., to monitor infection).

Family counseling and the early involvement of palliative care teams are recommended to determine the individual approach needed to ensure patient quality of life. Other interventions that can be discussed with families include nutritional support, physiotherapy, and spasticity management.

### 3.14. Other management and intervention issues

The diagnosis of ASMD can have major impacts on families and patients across the spectrum of disease phenotypes. Supportive services (social services, family counseling, patient and disease support groups) may be beneficial and can help optimize the management strategies necessary to ensure maximum quality of care and quality of life. Since emergency care physicians may not be familiar with chronic ASMD, emergency cards may be helpful as proposed by patient organization and Orphanet emergency guidelines for individuals with rare diseases [40]. Genetic counseling is important to educate patients and families on the autosomal recessive inheritance pattern, carrier status, and potential impact on future offspring and siblings. Carrier testing for at-risk relatives and prenatal diagnosis are possible if both *SMPD1* pathogenic variants in a family are identified. Prenatal diagnosis is also possible by testing for ASM enzyme activity.

Assessments and management of patients with chronic ASMD should take into account comorbid diseases that may present in patients as new information becomes available. For example, Hashimoto thyroiditis has been identified in several patients with ASMD, and some *SMPD1* mutations may be associated with an increased risk of Parkinson’s disease, although the pathogenic relationship between ASMD and Parkinson’s disease is not clear [41,42].

Finally, as ERT and other disease-specific therapies are developed for ASMD, and as natural history data grow more robust, additional options for assessing disease progression in patients will become appropriate. Currently, there are assessments typically made for research purposes that are not used in routine clinical monitoring that are worthy of mention. Imaging for assessment of liver and spleen volume may be made periodically to monitor disease progression and treatment responses. MRI or computed tomography are the recommended methods to calculate organ volume, although ultrasonography may be substituted in patients unable to tolerate the MRI or CT. Biomarker assays may be useful for disease monitoring and to monitor treatment responses once ERT or other disease-specific treatments become available, and are in the research stage of development [10,43]. Potential biomarkers for ASMD have been previously summarized [8], and recent studies have assessed the reliability of lysosphingomyelin [44] and lysosphingolipids [45] as biomarkers for diagnosis.

## 4. Conclusions

Current monitoring of ASMD is targeted towards reducing the impact of individual symptoms, with treatments, interventions, and lifestyle modifications designed to address morbidity and disease complications and improve patient quality of life. While patient monitoring must be performed based on the individual clinical profile, the proposed assessments and interventions in this paper evolved from review of the literature and the practices used by the authors in managing their own patients with ASMD. There is currently no disease-specific treatment for ASMD, although ERT with olipudase alfa is in clinical development [10]. Patients with chronic visceral and chronic neurovisceral ASMD require appropriate management by an interdisciplinary clinical team throughout childhood and adulthood. Consultation and communication among team members is essential for management of the multisystem manifestations of ASMD and for optimizing outcomes.

## Figures and Tables

**Table 1:** Characteristics of ASMD Subtypes (from [4]).

Most severe		Least severe
Infantile neurovisceral ASMD (NPD A)	Chronic neurovisceral ASMD (Intermediate; NPD A/B, NPD B variant)	Chronic visceral ASMD (NPD B)
Homogeneous natural history, life expectancy, and cause of death; Onset in early infancy (hepatosplenomegaly 2–4 months; neurological symptoms/developmental arrest between 6 and 12 months; hypotonia; ophthalmological changes); Rapidly progressive clinical manifestations including failure to thrive, neurodegeneration bleeding, respiratory infections secondary to aspiration; Death by 3 years of age	Onset in childhood with prolonged survival distinguishing it from infantile neurovisceral ASMD; Slower progression of neurological degeneration; Neurological symptoms variable (mild hypotonia/hyporeflexia due to loss of motor function and cognitive decline); Multisystem disease manifestations same as those for chronic visceral ASMD; Premature death during childhood or adulthood can occur from liver and/or respiratory disease	Variable age of onset ranging from infancy to adulthood; Slow and variable disease progression without neurodegeneration; Common clinical features include hepatosplenomegaly, proatherogenic lipid profile, delayed growth and puberty; complications include thrombocytopenia, slowly progressing interstitial lung disease, skeletal involvement, liver disease/cirrhosis; Patients may have a normal lifespan or die prematurely from complications including respiratory disease, liver disease, and hemorrhage

**Table 2:** Schedule of clinical assessments for patients with chronic visceral and chronic neurovisceral ASMD.

	Children	Adults
Initial/baseline assessments typically performed prior to/at diagnosis [see reference 7 for detailed information]	All patients: Growth measurements, liver function tests, liver and spleen size, ophthalmology exam, hematology, pulmonary function and lung imaging, cardiac assessments, skeletal radiographs, neurological, cognitive, and developmental assessments	All patients: Liver function tests, liver and spleen size, hematology, pulmonary function and lung imaging, cardiac assessments, coronary artery status (CT-scan), bone density, neurological assessments
	On an individual basis: Liver biopsy, portal pressure	On an individual basis: Liver biopsy, portal pressure, coronary catheterization
Every 3–6 months (depending on patient age)	Growth measurements, auscultation, age appropriate neurological and developmental assessments	
Annual assessments	Growth measurements, liver function, lipid profiles, auscultation, ECG, hematology, pulmonary function, age appropriate neurological and developmental assessments, hormone assessment depending on patient age, vaccines as needed	Liver function, portal hypertension, lipid profiles, auscultation, ECG, hematology, pulmonary function, peripheral neuropathy assessment, neuro psychology assessment, vaccines as needed
Periodic assessments (every 2-–4 years)	Echocardiogram, assess skeletal health using guidance provided by the International Society for Clinical Densitometry	Echocardiogram, lung imaging, coronary artery status, assess skeletal health using guidance provided by the International Society for Clinical Densitometry

See text and boxes for additional guidance on assessments. This schedule of assessments addresses key ASMD-related disease manifestations to monitor over the course of the disease in childhood and adulthood. The actual frequency of assessments should be decided by clinical teams according to each patient’s need for medical care and routine follow-up monitoring.

## Hepatic Assessments

- Periodic liver panels (every 6–12 months): transaminases, GGT, coagulation, albumin
- Monitor hepatic fibrosis (using transient elastography assessment of liver stiffness)
- Screen for esophageal varices in patients with portal hypertension based on clinical and radiological assessments (invasive and noninvasive assessments available)
- Monitor liver disease using Child-Pugh/MELD classification (note limitations)

Treatment/interventions/lifestyle modifications

- Maintain nutrition and control fluid retention
- Avoid alcohol use and hepatotoxic medications
- Vaccinations against viral hepatitis A and B
- Non-selective beta blockers for prevention of hemorrhage in patients with esophageal varices
- Ammonia reduction for hepatic encephalopathy
- Antibiotics as appropriate for spontaneous bacterial peritonitis
- Assessment of candidacy for liver transplant when needed

## Spleen assessments

Treatment/interventions/lifestyle modifications

- Since trauma-induced rupture is a possibility, caution regarding contact sports is advised

## Box

Pulmonary assessments

- Respiratory status assessed at every clinic visit
- Pulmonary function and diffusion capacity of lungs, and exercise tolerance should be assessed yearly or based on changes in patients’ clinical condition
- Chest radiographs should be obtained upon diagnosis with subsequent imaging (HRCT and X ray) every 2–4 years to monitor infiltrative lung disease

Treatment/interventions/lifestyle modifications

- Supplemental oxygen and/or noninvasive positive pressure ventilation should be prescribed based on underlying abnormalities such as hypoxemia and hypoventilation
- Bronchodilators may be used for symptomatic pulmonary disease
- Vaccinations (influenza, *pneumococcal pneumonia, haemophilus influenzae* Type B) as appropriate for patients with underlying lung disease
- All pulmonary infections should be aggressively managed
- Avoid exposure to tobacco products

## Cardiovascular Assessments

- Auscultation at every clinic visit
- Annual ECG
- Coronary artery status beginning at age 18 (HRCT scans be combined with pulmonary assessment every 2–4 years)
- Echocardiogram every 2–4 years
- Annual lipid profile

Treatment/interventions/lifestyle changes

- For dyslipidemia, diet management and possibly statins (post-puberty) (monitor liver function if statins are used) according to standard guidelines
- Medications for managing valvular insufficiency according to standard guidelines
- Surgery to repair/replace heart valves
- Stenting/CABG for coronary artery narrowing and blockages

## Hematology Assessments

- Complete blood counts annually or as indicated by findings
- Coagulation profile annually

Treatment/interventions/lifestyle modifications

- Interventions (e.g., packing, cauterization) for frequent or excessive nose bleeds

## Skeletal and Growth Assessments

- Monitor growth and weight gain in children at least every 6–12 months
- Monitor hormone levels for delayed puberty
- Skeletal health in children from infancy to adolescence and adults should be assessed using the guidance provided by the International Society for Clinical Densitometry

Treatment/interventions/lifestyle modifications

- Exercise to prevent osteopenia
- Physical therapy as needed
- Standard dietary and lifestyle interventions to minimize bone loss
- Ensure calorie intake is adequate for growth

## Neurological and Cognitive Assessments

- Annual neurological and developmental assessments in children (more frequently in younger patients)
- Ophthalmology exam at baseline
- More frequent monitoring of patients with the Q292K* mutation
- Peripheral neuropathy assessments annually
- Neuropsychology assessments as needed

Treatment/interventions/lifestyle modifications

- Educational support
- Physical therapy

*also referred to as Q294K.
